# Supplementary material for: Comorbidity clusters and in-hospital outcomes in patients admitted with acute myocardial infarction in the USA: A national population-based study
Source: PLoS One. 2023 Oct 26;18(10):e0293314. doi: 10.1371/journal.pone.0293314 (PMC10602297; doi:10.1371/journal.pone.0293314)
Supplement: S1 Fig — Radar charts present the percentage proportion of patients per latent class starting from 0% (chart centre). (PDF) [file pone.0293314.s001.pdf]

Figure S1 – Latent class membership (percentage) by age, sex, age/sex, and race

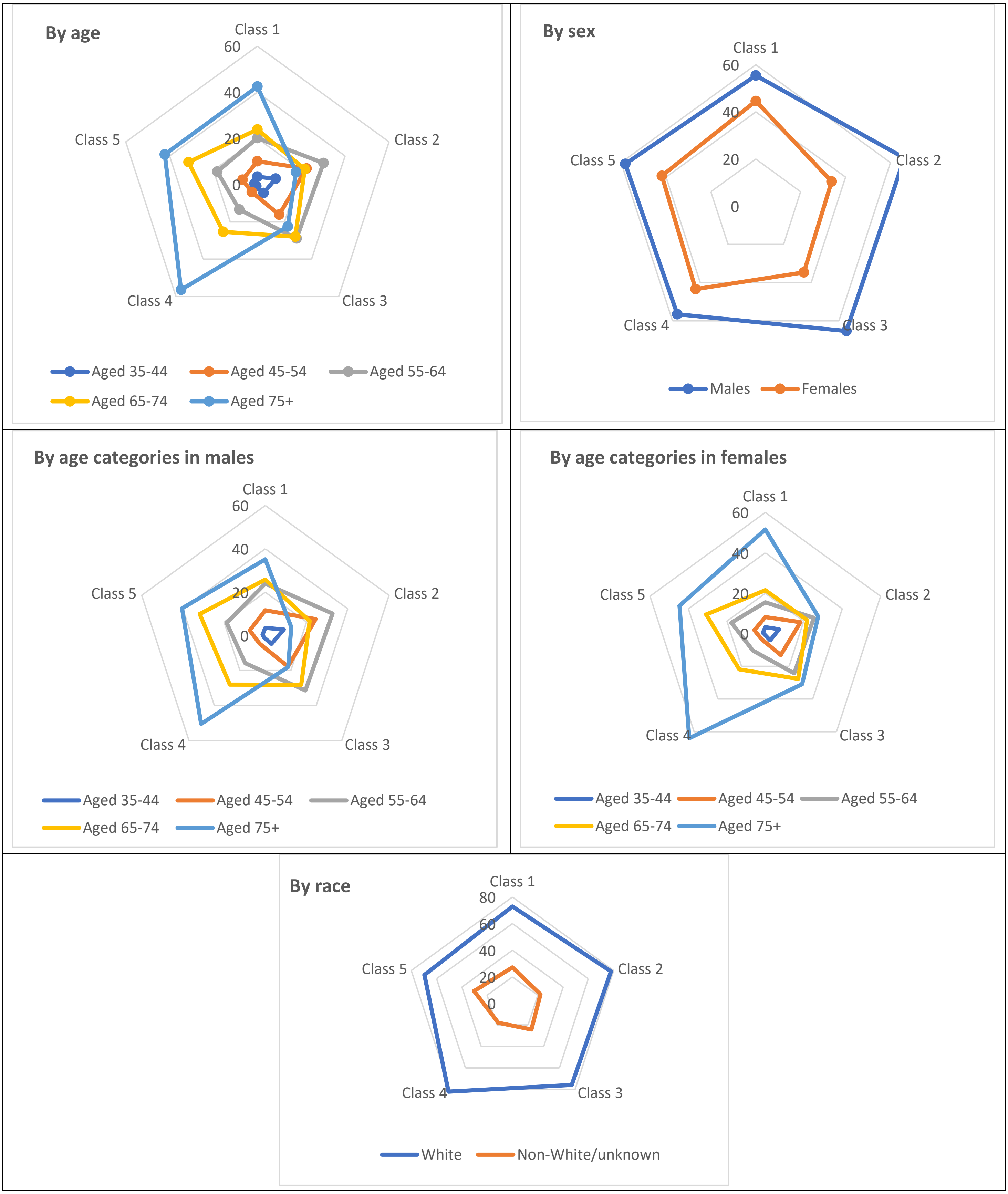

Radar charts present the percentage proportion of patients per latent class starting from 0% (chart centre)
