# Supplementary material for: Comorbidity clusters and in-hospital outcomes in patients admitted with acute myocardial infarction in the USA: A national population-based study
Source: PLoS One. 2023 Oct 26;18(10):e0293314. doi: 10.1371/journal.pone.0293314 (PMC10602297; doi:10.1371/journal.pone.0293314)

*Supplemental Material to 'Comorbidity clusters and in-hospital outcomes in patients admitted with AMI in the USA' Zghebi SS et al. 2023*

## Figure S2 – Individual radar charts for percentage prevalence of comorbidities per latent class

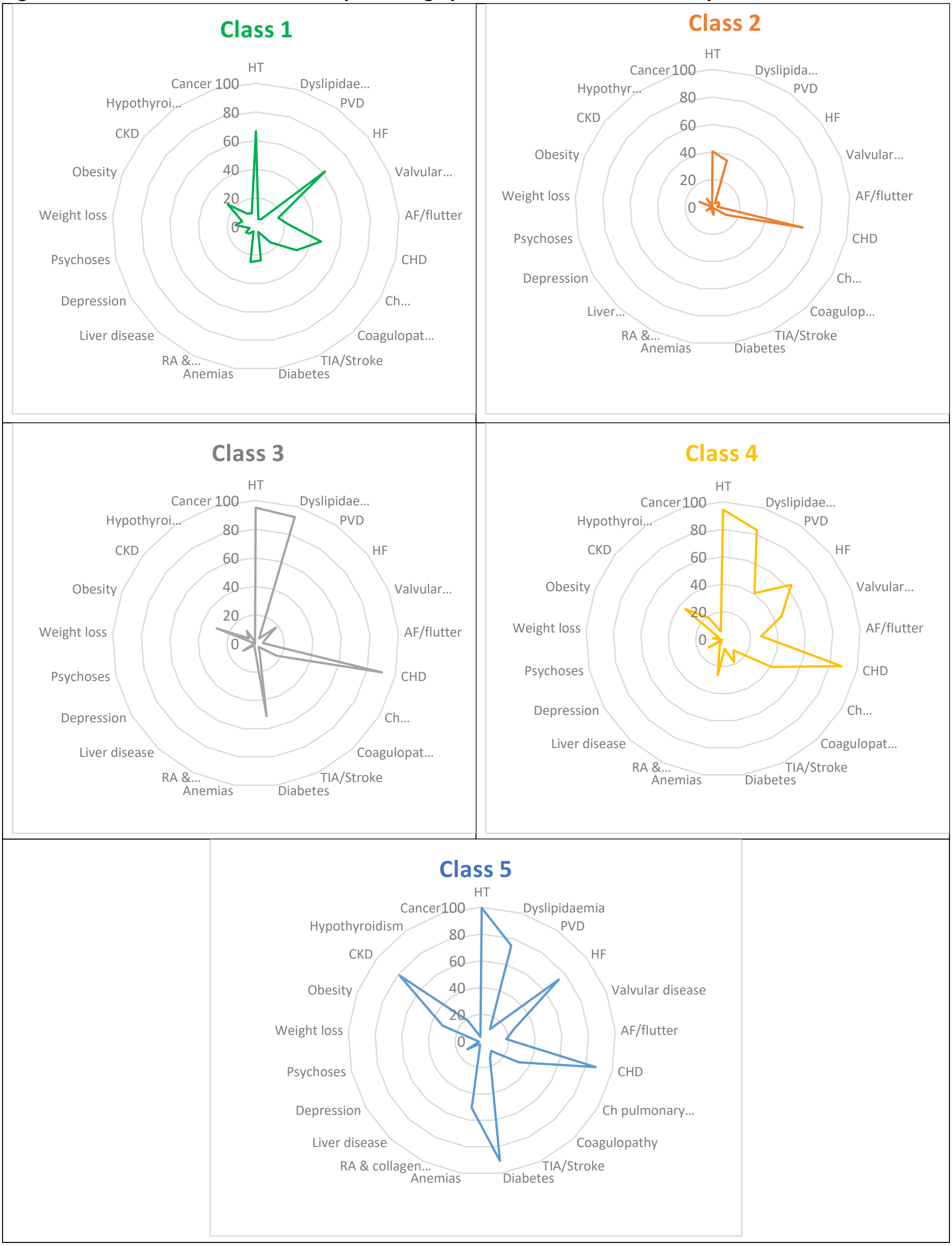

Supplement: S2 Fig — (PDF) [file pone.0293314.s002.pdf]
