# Supplementary figures and images for: Comorbidity clusters and in-hospital outcomes in patients admitted with acute myocardial infarction in the USA: A national population-based study
Source: PLoS One. 2023 Oct 26;18(10):e0293314. doi: 10.1371/journal.pone.0293314 (PMC10602297; doi:10.1371/journal.pone.0293314)

Figure S3 – Radar charts for percentage prevalence of in-hospital outcomes per latent class

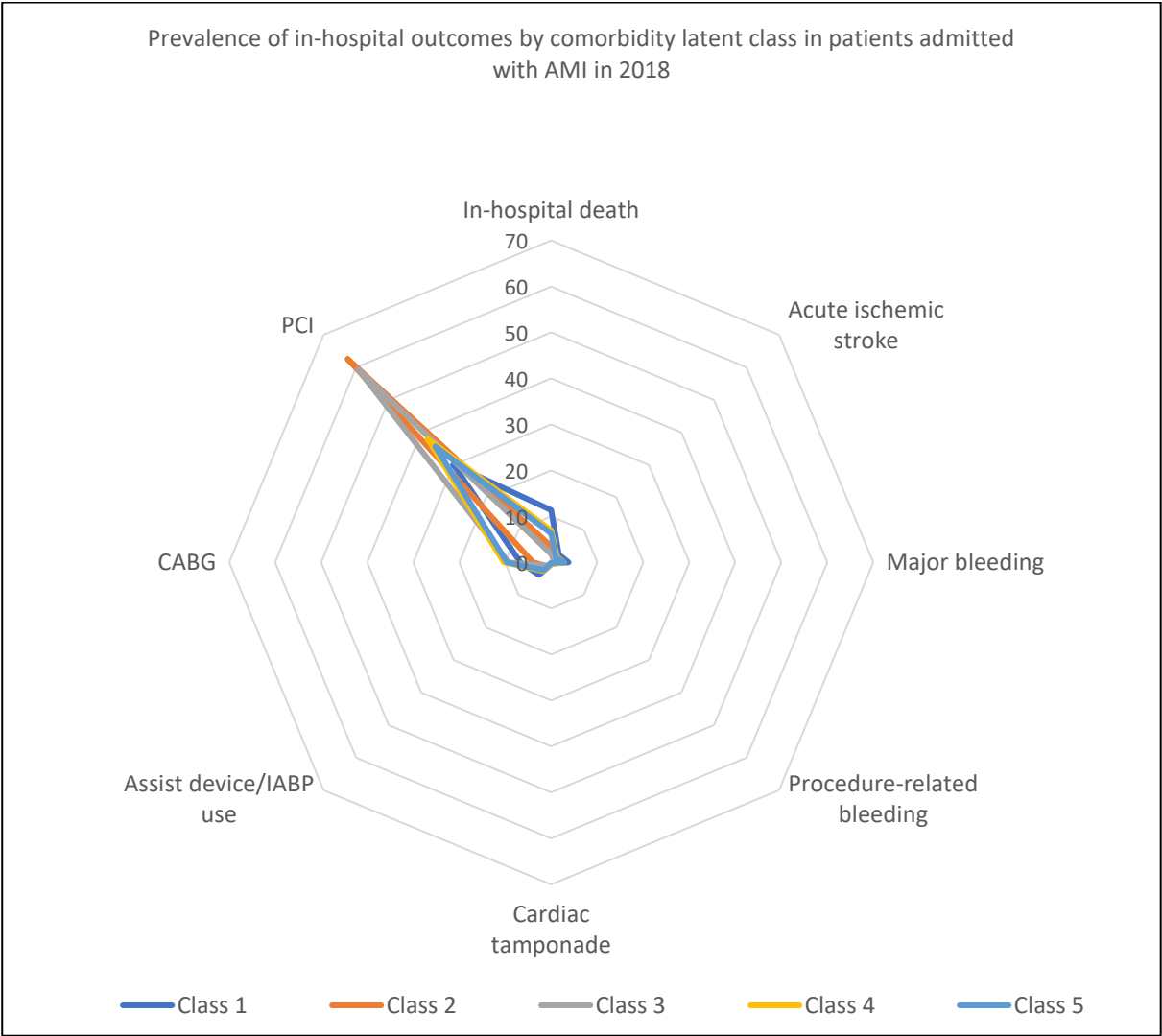

Supplement: S3 Fig — (PDF) [file pone.0293314.s003.pdf]

**Figure S4 Predictive margins (95% CI) of probabilities of outcomes per latent class**

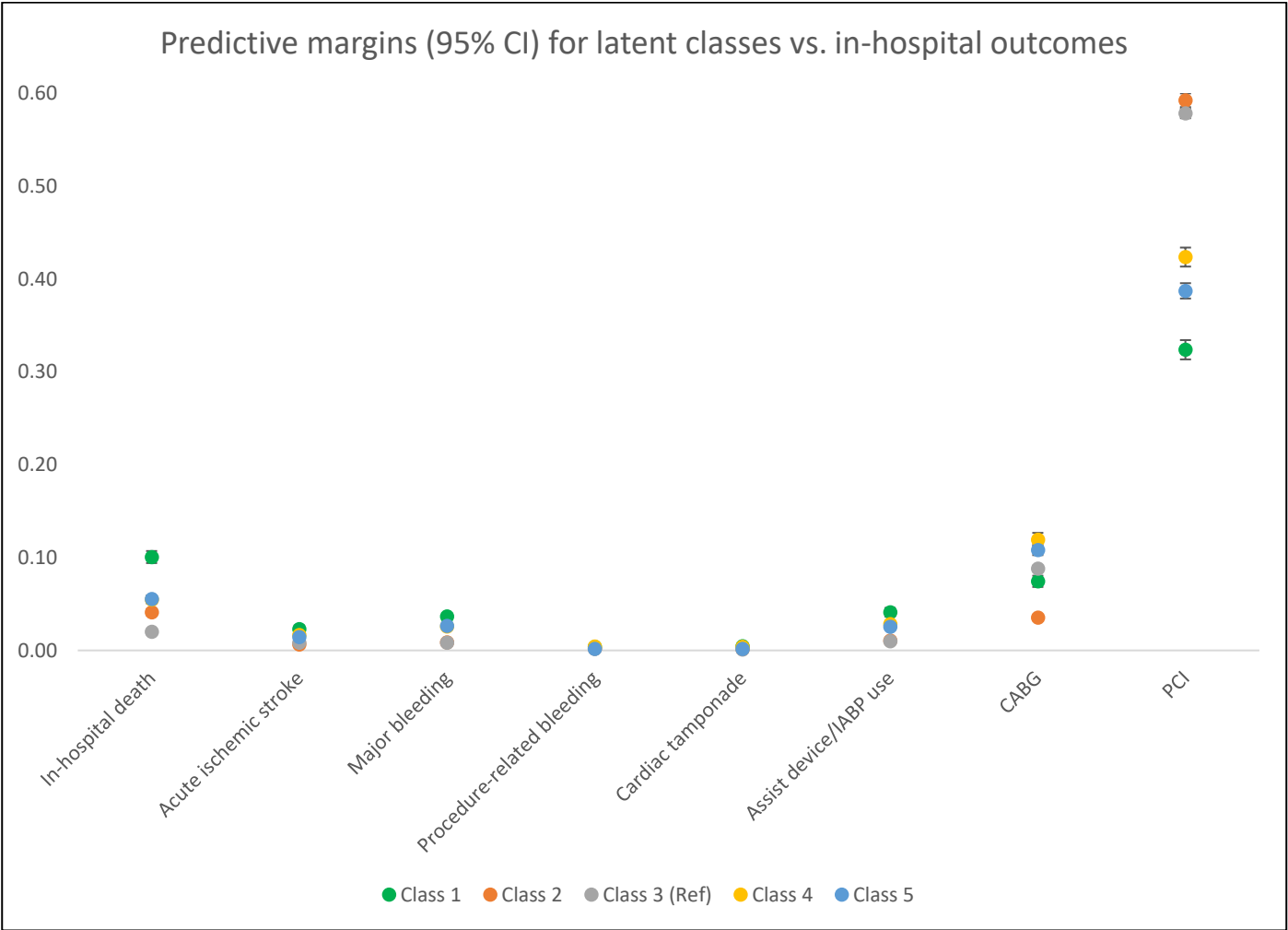

Supplement: S4 Fig — (PDF) [file pone.0293314.s004.pdf]
