# Supplementary material for: Comorbidity clusters and in-hospital outcomes in patients admitted with acute myocardial infarction in the USA: A national population-based study
Source: PLoS One. 2023 Oct 26;18(10):e0293314. doi: 10.1371/journal.pone.0293314 (PMC10602297; doi:10.1371/journal.pone.0293314)
Supplement: S1 Table — (PDF) [file pone.0293314.s005.pdf]

Table S1 ICD-10 and procedure codes for outcomes

Outcome	Codes
**Acute ischemic stroke**	I63*
**Major bleeding (including haemorrhagic stroke)**	I60* I61* I62* (Haemorrhagic strokes) R58 K920 K921 K922
**Procedure-related bleeding**	I97410 I97610 I97411 I97611
**Cardiac tamponade**	I314
**Assist device/IABP use**	5A02110 5A0211D 5A02216 02HA3RJ 02HA3RZ
**CABG**	0210083 0210088 0210089 021008C 021008W 02100J3 02100J8 02100J9 02100JC 02100JW 02100K3 02100K8 02100K9 02100KC 02100KW 02100Z3 02100Z8 02100Z9 02100ZC 0210444 0210483 0210488 0210489 021048C 021048W 02104D4 02104J3 02104J8 02104J9 02104JC 02104JW 02104K3 02104K8 02104K9 02104KC 02104KW 02104Z3 02104Z8 02104Z9 02104ZC 0211083 0211088 0211089 021108C 021108W 02110J3 02110J8 02110J9 02110JC 02110JW 02110K3 02110K8 02110K9 02110KC 02110KW 02110Z3 02110Z8 02110Z9 02110ZC 0211444 0211483 0211488 0211489 021148C 021148W 02114D4 02114J3 02114J8 02114J9 02114JC 02114JW 02114K3 02114K8 02114K9 02114KC 02114KW 02114Z3 02114Z8 02114Z9 02114ZC 0212083 0212088 0212089 021208C 021208W 02120J3 02120J8 02120J9 02120JC 02120JW 02120K3 02120K8 02120K9 02120KC 02120KW 02120Z3 02120Z8 02120Z9 02120ZC 0212444 0212483 0212488 0212489 021248C 021248W 02124D4 02124J3 02124J8 02124J9 02124JC 02124JW 02124K3 02124K8 02124K9 02124KC 02124KW 02124Z3 02124Z8 02124Z9 02124ZC 0213083 0213088 0213089 021308C 021308W 02130J3 02130J8 02130J9 02130JC 02130JW 02130K3 02130K8 02130K9 02130KC 02130KW 02130Z3 02130Z8 02130Z9 02130ZC 0213444 0213483 0213488 0213489 021348C 021348W 02134D4 02134J3 02134J8 02134J9 02134JC 02134JW 02134K3 02134K8 02134K9 02134KC 02134KW 02134Z3 02134Z8 02134Z9 02134ZC
**PCI**	0270346 027034Z 02703D6 02703DZ 02703T6 02703TZ 02703Z6 02703ZZ 027044Z 0270446 0270356 027035Z 02703E6 02703EZ 0270456 027045Z 0270366 027036Z 02703F6 02703FZ 0270466 027046Z 0270376 027037Z 02703D6 02703DZ 027047Z 0270476 0271346 027134Z 02713D6 02713DZ 02713T6 02713TZ 02713Z6 02713ZZ 0271356 027135Z 02713E6 02713EZ 0271366 027136Z 02713F6 02713FZ 0271376 027137Z 02713G6 02713GZ 0272346 027234Z 02723D6 02723DZ 02723T6 02723TZ 02723Z6 02723ZZ 0272356 027235Z 02723E6 02723EZ 0272366 027236Z 02723F6 02723FZ 0272376 027237Z 02723G6 02723GZ 0273346 027334Z 02733D6 02733DZ 02733T6 02733TZ 02733Z6 02733ZZ 0273356 027335Z 02733E6 02733EZ 0273366 027336Z 02733F6 02733FZ 0273376 027337Z 02733G6 02733GZ
