# Supplementary material for: Comorbidity clusters and in-hospital outcomes in patients admitted with acute myocardial infarction in the USA: A national population-based study
Source: PLoS One. 2023 Oct 26;18(10):e0293314. doi: 10.1371/journal.pone.0293314 (PMC10602297; doi:10.1371/journal.pone.0293314)
Supplement: S2 Table — (PDF) [file pone.0293314.s006.pdf]

Table S2 Comparison of AIC/BIC values between models

Number of classes	AIC	BIC
2	1263217	1263618
3	1253866	1254473
4	1249884	1250696
5	1247500	1248517
6	1246092	1247315
7	Convergence not achieved	Convergence not achieved
8	Convergence not achieved	Convergence not achieved
