# Supplementary material for: Comorbidity clusters and in-hospital outcomes in patients admitted with acute myocardial infarction in the USA: A national population-based study
Source: PLoS One. 2023 Oct 26;18(10):e0293314. doi: 10.1371/journal.pone.0293314 (PMC10602297; doi:10.1371/journal.pone.0293314)
Supplement: S3 Table — (PDF) [file pone.0293314.s007.pdf]

Table S3 Significance testing for baseline characteristics and comorbidity burden across latent classes

Comorbidity latent classes – Nominal (exploratory) P-values are reported.<br><br>Among all classes, patients in Class 1 had the lowest prevalence of dyslipidemia (p<0.001) and CHD (p<0.001), patients in Class 2 had no CKD and broadly lowest prevalence of all comorbidities except for CHD, RA, mental illness & obesity, while Class 4 had lowest prevalence of obesity (p<0.001) and psychoses (p<0.001). Patients in Class 1 had highest prevalence of cancer (p<0.001), weight loss (p<0.001), psychoses (p<0.001), liver disease (p<0.001), and coagulopathy (p<0.001) compared to other classes; members of Class 3 had highest prevalence of CHD and dyslipidemia; Class 4 had highest prevalence of TIA/stroke (p <0.001), chronic pulmonary disease (p<0.001), AF/flutter (p<0.001), valvular disease (p <0.001) and PVD (p<0.001); patients in Class 5 had least proportion of White race (70%), had the overall highest comorbidity burden, specifically the highest prevalence of HTN (99.6%, p<0.001), CKD (p<0.001), obesity (p<0.001), anaemias (p<0.001), diabetes (p<0.001), and HF (p<0.001) compared to patients in other classes (Table 1 and Figure 1).<br>Overall, mean age (±SD) was 67 (±13) years, 38% were females, 12% Black, and 43% admitted in Southern hospitals (Table 2). Patients in Class 2 were the youngest (age 62±13 years, p<0.001) and had the highest male proportion (66%, p<0.001), and the lowest hospital costs ($59,855 (IQR 57,402), p<0.001) compared to patients in other classes (Table 2). On the other hand, Class 4 included the oldest patients (75±11 years, p<0.001) and fewest Hispanic (2.9%, p<0.001) among all patients admitted with AMI (Table 2 and Figure S2). By both age and gender, 64% of females vs. 51% of males in Class 4 were aged 75+years (Figure S2). Patients in Class 2 and Class 3 spent 2 (IQR 2) days in hospital, while Class 5 had the longest hospital spells of 5(IQR 5) days (p<0.001).
