# Supplementary material for: Comorbidity clusters and in-hospital outcomes in patients admitted with acute myocardial infarction in the USA: A national population-based study
Source: PLoS One. 2023 Oct 26;18(10):e0293314. doi: 10.1371/journal.pone.0293314 (PMC10602297; doi:10.1371/journal.pone.0293314)
Supplement: S4 Table — (PDF) [file pone.0293314.s008.pdf]

Table S4 Crude rates of in-hospital outcomes in patients admitted with AMI in 2018

	**Overall**	**Class 1** (Cancer/ coagulopathy/ liver)	**Class 2** (Least burdened)	**Class 3** (CHD/ dyslipidemia)	**Class 4** (COPD/VD/PVD)	**Class 5** (DM/CKD/HF)
**In-hospital death, %**	4.4	11.3	3.5	1.8	7.0	6.3
**Major bleeding, %**	1.7	3.8	0.8	0.8	2.8	2.8
**Acute ischemic stroke, %**	1.1	2.4	0.6	0.8	1.7	1.5
**Procedure-related bleeding, %**	0.2	0.4	0.2	0.2	0.4	0.2
**Cardiac tamponade, %**	0.2	0.4	0.1	0.1	0.4	0.2
**Assist device/IABP use, %**	1.7	3.8	1.1	1.0	2.6	2.4
**CABG, %**	8.1	6.7	4.0	9.3	10.3	9.6
**PCI, %**	50.8	30.1	62.5	59.4	38.0	35.6
