# Supplementary material for: Comorbidity clusters and in-hospital outcomes in patients admitted with acute myocardial infarction in the USA: A national population-based study
Source: PLoS One. 2023 Oct 26;18(10):e0293314. doi: 10.1371/journal.pone.0293314 (PMC10602297; doi:10.1371/journal.pone.0293314)
Supplement: S5 Table — Includes haemorrhagic stroke. ^ Class 3 is the largest class and was selected as the reference group. $ The ‘Other’ race group for these outcomes includes Hispanic, Asian/Pacific Islander, Native American and ‘Other’ categories. CABG: coronary artery bypass graft; CHD: coronary heart disease; DM: diabetes; CKD: chronic kidney disease; COPD: chronic obstructive pulmonary disease; HF: heart failure; IABP: intra-aortic balloon pump; PCI: percutaneous coronary intervention; PVD: peripheral vascular disease; VD: valvular disease. (PDF) [file pone.0293314.s009.pdf]

Table S5 Odds ratios (95% CI) for risks of in-hospital outcomes in patients admitted with AMI in 2018

	In-hospital death	Major bleeding*	Acute ischemic stroke	Procedure-related bleeding	Cardiac tamponade	Use of assist device/IABP	CABG	PCI
**Age**	1.04 (1.03; 1.04)	1.01 (1.01; 1.01)	1.01 (1.00; 1.01)	1.00 (0.99; 1.01)	0.99 (0.98; 1.00)	0.99 (0.99; 1.00)	0.98 (0.98; 0.98)	0.98 (0.98; 0.98)
**Sex** (Female)	0.98 (0.92; 1.05)	0.92 (0.82; 1.03)	1.17 (1.03; 1.34)	1.09 (0.81; 1.46)	0.97 (0.69; 1.35)	0.63 (0.56; 0.71)	0.59 (0.55; 0.62)	0.77 (0.75; 0.79)
**Race**<br>White	Ref	Ref	Ref	Ref	Ref	Ref	Ref	Ref
Black	1.06 (0.95; 1.18)	1.11 (0.94; 1.31)	1.24 (1.02; 1.49)	1.07 (0.68; 1.69)	0.67 (0.38; 1.18)	0.65 (0.53; 0.78)	0.63 (0.57; 0.68)	0.67 (0.64; 0.70)
Hispanic	0.96 (0.81; 1.14)	1.14 (0.90; 1.46)	1.10 (0.82; 1.48)	-	-	1.03 (0.81; 1.31)	0.93 (0.82; 1.04)	0.84 (0.78; 0.89)
Asian/Pacific Islander	1.38 (1.07; 1.79)	1.09 (0.70; 1.71)	1.15 (0.67; 1.95)	-	-	1.11 (0.73; 1.68)	1.05 (0.86; 1.28)	1.04 (0.92; 1.17)
Native American	1.30 (0.75; 2.25)	1.30 (0.57; 2.90)	0.62 (0.15; 2.50)	-	-	0.55 (0.17; 1.73)	0.98 (0.65; 1.46)	0.83 (0.65; 1.05)
Other	1.44 (1.18; 1.75)	0.96 (0.67; 1.38)	1.46 (1.02; 2.09)	1.52 (0.99; 2.35)[$]	1.07 (0.63; 1.83)[$]	1.56 (1.20; 2.07)	0.80 (0.68; 0.95)	1.06 (0.97; 1.16)
Unknown	1.72 (1.46; 2.03)	1.60 (1.23; 2.08)	1.24 (0.87; 1.76)	1.10 (0.48; 2.48)	0.63 (0.20; 1.97)	1.80 (1.42; 2.28)	1.24 (1.09; 1.42)	1.14 (1.05; 1.23)
**Latent class**	5.57 (4.99; 6.21)	4.48 (3.78; 5.31)	2.76 (2.27; 3.35)	2.09 (1.33; 3.28)	4.28 (2.64; 6.94)	4.33 (3.67; 5.09)	0.83 (0.75; 0.91)	0.34 (0.32; 0.36)

Class 1 (Cancer/ coagulopathy /liver)								
Class 2 (Least burdened)	2.11 (1.89; 2.37)	1.04 (0.85; 1.27)	0.75 (0.60; 0.94)	1.09 (0.71; 1.65)	1.18 (0.70; 2.01)	1.08 (0.90; 1.28)	0.38 (0.34; 0.41)	1.06 (1.02; 1.10)
Class 3^ (CHD/dyslipidemia)	Ref	Ref	Ref	Ref	Ref	Ref	Ref	Ref
Class 4 (COPD/VD/PVD)	2.85 (2.54; 3.21)	3.14 (2.63; 3.74)	2.00 (1.63; 2.46)	2.25 (1.48; 3.44)	3.70 (2.27; 6.02)	2.95 (2.47; 3.52)	1.41 (1.30; 1.53)	0.53 (0.50; 0.55)
Class 5 (DM/CKD/HF)	2.89 (2.60; 3.22)	3.20 (2.73; 3.75)	1.71 (1.42; 2.06)	0.94 (0.58; 1.53)	1.58 (0.93; 2.70)	2.63 (2.24; 3.08)	1.26 (1.17; 1.35)	0.45 (0.43; 0.47)

* Includes haemorrhagic stroke.
^ Class 3 is the largest class and was selected as the reference group.
$ The 'Other' race group for these outcomes includes Hispanic, Asian/Pacific Islander, Native American and 'Other' categories.

CABG: coronary artery bypass graft; CHD: coronary heart disease; DM: diabetes; CKD: chronic kidney disease; COPD: chronic obstructive pulmonary disease; HF: heart failure; IABP: intra-aortic balloon pump; PCI: percutaneous coronary intervention; PVD: peripheral vascular disease; VD: valvular disease.
