# Supplementary material for: Comorbidity clusters and in-hospital outcomes in patients admitted with acute myocardial infarction in the USA: A national population-based study
Source: PLoS One. 2023 Oct 26;18(10):e0293314. doi: 10.1371/journal.pone.0293314 (PMC10602297; doi:10.1371/journal.pone.0293314)
Supplement: S6 Table — ^ Class 3 is the largest class and was selected as the reference group. CHD: coronary heart disease; DM: diabetes; CKD: chronic kidney disease; COPD: chronic obstructive pulmonary disease; HF: heart failure; PVD: peripheral vascular disease; VD: valvular disease. (PDF) [file pone.0293314.s010.pdf]

Table S6 Regression coefficients (95% CI) of predictors of hospital costs in patients admitted with AMI in 2018

	**Coefficient (95% CI)**
**Age**	-747.7 (-812.6; -682.9)
**Sex** (Female)	-13976.0 (-15508.2; -12443.8)
**Race**<br>White	Ref
Black	-7156.1 (-9523.1; -4789.2)
Hispanic	21165.6 (17148.0; 25183.2)
Asian/Pacific Islander	10857.5 (3897.1; 17817.9)
Native American	-20420.9 (-32323.8; -8518.0)
Other	11551.0 (6209.9; 16892.1)
Unknown	10262.7 (4765.9; 15759.4)
**Latent class**<br>Class 1 (Cancer/ coagulopathy /liver)	41205.5 (36345.9; 46065.0)
Class 2 (Least burdened)	-13369.3 (-14752.2; -11986.4)
Class 3^ (CHD/dyslipidemia)	Ref
Class 4 (COPD/VD/PVD)	26298.3 (23530.6; 29066.1)
Class 5 (DM/CKD/HF)	139261.5 (134859.9; 143663.0)

^ Class 3 is the largest class and was selected as the reference group

CHD: coronary heart disease; DM: diabetes; CKD: chronic kidney disease; COPD: chronic obstructive pulmonary disease; HF: heart failure; PVD: peripheral vascular disease; VD: valvular disease.
