# Supplementary material for: Comorbidity clusters and in-hospital outcomes in patients admitted with acute myocardial infarction in the USA: A national population-based study
Source: PLoS One. 2023 Oct 26;18(10):e0293314. doi: 10.1371/journal.pone.0293314 (PMC10602297; doi:10.1371/journal.pone.0293314)
Supplement: S7 Table — ^ Class 3 is the largest class and was selected as the reference group. CHD: coronary heart disease; DM: diabetes; CKD: chronic kidney disease; COPD: chronic obstructive pulmonary disease; HF: heart failure; PVD: peripheral vascular disease; VD: valvular disease. (PDF) [file pone.0293314.s011.pdf]

Table S7 Incidence rate ratios (IRRs) (95% CI) of predictors of length of hospital stay in patients admitted with AMI in 2018

	**IRR (95% CI)**
**Age**	1.00 (1.00; 1.00)
**Sex** (Female)	0.95 (0.93; 0.96)
**Race**<br>White	Ref
Black	1.04 (1.01; 1.06)
Hispanic	1.10 (1.06; 1.13)
Asian/Pacific Islander	1.06 (1.00; 1.13)
Native American	0.93 (0.79; 1.10)
Other	1.06 (1.02; 1.11)
Unknown	1.01 (0.97; 1.06)
**Latent class**<br>Class 1 (Cancer/ coagulopathy /liver)	1.86 (1.81; 1.92)
Class 2 (Least burdened)	0.79 (0.77; 0.80)
Class 3^ (CHD/dyslipidemia)	Ref
Class 4 (COPD/VD/PVD)	1.61 (1.57; 1.65)
Class 5 (DM/CKD/HF)	1.80 (1.77; 1.84)

^ Class 3 is the largest class and was selected as the reference group

CHD: coronary heart disease; DM: diabetes; CKD: chronic kidney disease; COPD: chronic obstructive pulmonary disease; HF: heart failure; PVD: peripheral vascular disease; VD: valvular disease.
